# Effects of *Euglena gracilis* Intake on Mood and Autonomic Activity under Mental Workload, and Subjective Sleep Quality: A Randomized, Double-Blind, Placebo-Controlled Trial

**DOI:** 10.3390/nu12113243

**Published:** 2020-10-23

**Authors:** Ayaka Nakashima, Kosuke Yasuda, Ako Murata, Kengo Suzuki, Naoki Miura

**Affiliations:** 1 Euglena Co. Ltd., Tokyo 108-0014, Japan; kosuke.yasuda@euglena.jp (K.Y.); ako.murata@euglena.jp (A.M.); suzuki@euglena.jp (K.S.); 2 Miura Clinic, Medical Corporation Kanonkai, Osaka 530-0044, Japan; miura@miura-cl.jp

**Keywords:** *Euglena gracilis*, paramylon, autonomic nervous system

## Abstract

While the human body maintains homeostasis by altering the balance in the autonomic nervous, endocrine, and immune systems, a prolonged imbalance in these systems can result in physical and mental symptoms, including a decline in sleep quality and work efficiency. *Euglena* *gracilis* (*Euglena*) is a single-celled microalga with the properties of both plants and animals and contains abundant nutrients, such as vitamins, minerals, amino acids, and fatty acids, which have various beneficial health effects. This study evaluated the effects of *Euglena* intake on the mood states and stress coping under mental workload tasks, and subjective sleep quality. We assigned men and women aged 20 to 64 years to *Euglena* and placebo intake groups, and measured indices related to the autonomic nervous system, psychological states, and sleep quality together with the application of workload stress before food intake, and 4, 8, and 12 weeks after commencing intake. *Euglena* intake regulated the autonomic nervous system under a workload and improved psychological parameters and sleep conditions. These results indicate that the consumption of *Euglena* may regulate the balance of the autonomic nervous system during stress and may have a favorable effect on psychological status and sleep quality.

## 1. Introduction

Stress is a general term for the defense response to external stimuli, such as pain, cold, and infection, and can include mental tension and worry [1]. Recently, there has been considerable interest in the effects of psychological stress on the body, particularly in light of the discovery of a neural pathway that transmits stress signals from specific areas of the cerebral cortex, which processes psychological stress and emotions, to the hypothalamus, which controls the sympathetic nervous system [2]. The human body is constantly exposed to stress and works to maintain a constant internal environment via the control of the autonomic nervous, endocrine, and immune systems for protection [3]. However, excessive stress weakens the body’s stress resistance, resulting in physical and psychological symptoms, including a deterioration in the quality of sleep and reduced work efficiency [4].

The autonomic nervous system, which includes the sympathetic and parasympathetic nervous systems, plays several important roles in the regulation of biological functions, such as respiration/circulation, digestion/absorption, secretion, and metabolism in a coordinated manner to maintain homeostasis [5]. For example, an imbalance in the autonomic nervous system may prevent the activation of the sympathetic nervous system, even during activities when it is typically activated, or the activation of the parasympathetic nervous system, even during recovery and rest. These conditions lead to an inability to concentrate and various ailments, such as poor sleep quality, reduced immunity, poor bowel movements, as well as various diseases [5]. Eliminating the underlying stress is an effective approach to balancing the autonomic nervous system. However, adjustments in lifestyle and nutrition are also effective.

*Euglena gracilis* (*Euglena*) is a single-celled microalga with plant and animal properties. *Euglena* consists of various nutrients, such as vitamins, minerals, amino acids, and fatty acids, and are used as nutritional and general supplements [6]. Previous studies have confirmed that *Euglena* intake effectively suppresses elevated blood glucose levels [7], suppresses fat accumulation [8,9], attenuates lifestyle-related disease symptoms [10], promotes immune function [11,12], and improves bowel movements [13]. Autonomic nervous system activity plays an important role in maintaining homeostasis and is involved in biological processes such as lipolysis, blood glucose regulation, immune function, and digestion. For example, the autonomic nervous system of the pancreas contributes to the regulation of blood glucose levels by regulating insulin secretion [14]; in white adipose tissue the autonomic nervous system is involved in lipolysis [14,15]; in the spleen, it is involved in immunity [16,17], and in the stomach and intestines it is involved in the promotion of peristaltic movements [18]. These findings led us to hypothesize that various beneficial health effects of *Euglena* intake are mediated by its regulation of the autonomic nervous system balance.

In this study, we investigated the effects of the ingestion of *Euglena*-containing food for 12 weeks on the autonomic nervous system, psychological factors, and quality of sleep in men and women aged 20 to 64 years suffering from decreased motivation and a decline in sleep quality.

## 2. Materials and Methods

### 2.1. Materials

*Euglena gracilis* powder was obtained from euglena Co. Ltd. (Tokyo, Japan). The placebo food was the same formula but without *Euglena* powder.

### 2.2. Ingestion Study

#### 2.2.1. Target Population

The study was conducted between July to November 2019. Subjects were healthy men and women between the ages of 20 and 64 years who were presented with reduced motivation based on the vitality (VT) measured by 36-Item Short-Form Health Survey (SF-36) and poor quality of sleep based on the Pittsburgh Sleep Quality Index (PSQI) total score. The purpose and content of the study were thoroughly explained to the subjects both verbally and in writing, and the subjects consented to participation by providing written informed consent. This study was conducted in accordance with the tenets of the Declaration of Helsinki and the Ethical Guidelines for Medical and Health Research Involving Human Subjects. Protection of the human rights of the subjects was always ensured, and the study was implemented under the supervision of a doctor with the approval of the ethics review committee of the Miura Clinic (approval number: R1816, approval date: 27 June 2019). This trial is registered with UMIN ID: 000037726.

#### 2.2.2. Test Method

This study was designed and implemented as a double-blind placebo-controlled trial. Subjects were selected after screening based on the level of motivation and quality of sleep. After baseline (week 0) measurements were obtained before the first dose, and the subjects received 500, 1000, or 3000 mg of *Euglena* powder or placebo powder (starch), divided into two doses per day (after breakfast and after dinner) for 12 weeks. The same measurements were taken at 4, 8, and 12 weeks after commencing intake. Subjects were instructed not to eat or drink after 10:00 p.m. on the day prior to the test and not to consume anything other than water until the designated meal was consumed on test day. On the examination day, subjects consumed the designated meal (200 g of rice) at least 4 h before they visited the hospital and had only water until the end of the examination. On the day measurements were taken, the subjects underwent a subjective index survey using Visual Analog Scale (VAS), Profile of Mood States 2nd Edition (POMS2), SF-36, PSQI, Ogri-Shirakawa-Azumi Sleep Inventory MA Version (OSA-MS), autonomic system response indicator measurements, saliva collection, blood collection, and physical measurements such as height, weight and body mass index (BMI). After resting for 30 min, the subjects were subjected to the Uchida–Kraepelin [19,20,21,22] test for workload stress. The test took 30 min, including 15 min for the first half and 15 min for the second half with a break in the middle, while changing each line of simple single-digit addition once a minute. The correct answer rate for 15 min was calculated. The autonomic system response indicator measurements, saliva collection, and subjective index survey were repeated immediately after the Uchida–Kraepelin test. After resting for 60 min, the autonomic system response indicator measurements, saliva collection, and subjective index survey were performed again. During the test, the subjects drank water (about 100 mL) between the second subjective index survey and before starting the 60 min rest period.

##### Autonomic System Response Indicator Measurements

The balance of the autonomic nervous system, an indicator of fatigue and stress, was evaluated at three points for each test, i.e., before Uchida–Kraepelin stress loading, immediately after Uchida–Kraepelin stress loading, and 60 min after Uchida–Kraepelin stress loading. The tests were conducted before intake and at 4, 8, and 12 weeks after starting intake. The vital monitor VM-302 (Fatigue Science Laboratory Inc., Osaka, Japan) was used to simultaneously measure the pulse wave (PPG) and cardiac wave (ECG) signals, and to analyze high-frequency (HF) and low-frequency (LF) components of heart rate fluctuation [21]. HF reflects the parasympathetic response and LF reflects the sympathetic response.

##### Measurements of Health-Related Quality of Life through the 36-Item Short-Form Health Survey (SF-36)

The health of the subjects was surveyed with a quality of life questionnaire before intake and at 4, 8, and 12 weeks after starting intake using an SF-36 v2 [23,24,25], based on universally applicable health-related concepts. A five-point scale was used for responses to each of the 36 questions, and the questions included eight items; physical functioning (PF), role physical (RP), bodily pain (BP), general health (GH), vitality (VT), social functioning (SF), role emotional (RE), and mental health (MH).

##### Measurements of Mood States Using the Profile of Mood States 2nd Edition (POMS2)

Six items, anxiety and tension, hostility and anger, depression and discouragement, vitality and activity, fatigue and lethargy, and confusion and perplexity, were evaluated based on the T score using the Japanese version of the POMS2 [22,26,27] at three points for each test, before Uchida–Kraepelin stress loading, after Uchida–Kraepelin stress loading, and 60 min after Uchida–Kraepelin stress loading. Measurements were obtained before intake and at 4, 8, and 12 weeks after starting intake.

##### Visual Analog Scale (VAS)

The 100-mm VAS was used to quantify fatigue, irritability, concentration, mood, motivation, tension, and satisfaction with sleep for 1 week at three points for each test, before Uchida–Kraepelin stress loading, after Uchida–Kraepelin stress loading, and 60 min after Uchida–Kraepelin stress loading, before intake and at 4, 8, and 12 weeks after starting intake. Each VAS question had an endpoint of 0 indicating a “positive situation” and an endpoint of 100 indicating a “negative situation.” Subjects were asked to mark the position on the line that represented their mood at the time. The distance from the endpoint of 0 to the mark was measured in mm.

##### Measurements of Quality of Sleep Using the Ogri-Shirakawa-Azumi Sleep Inventory MA Version (OSA-MA)

The sleep profile of the subjects was confirmed using OSA-MA [28,29], which includes five items related to sleep: sleepiness on rising, initiation and maintenance of sleep, frequent dreaming, refreshing, and sleep length. Items were assessed before intake and at 4, 8, and 12 weeks after starting intake. As for the score polarity, the direction of the good feeling of sleep had the highest score.

##### Pittsburgh Sleep Quality Index (PSQI)

The Japanese versionof the PSQI (PSQI-J) [29,30] was used to evaluate sleep quality before intake and at 4, 8, and 12 weeks after starting intake. The questions refer to the subject’s sleep profile in the past month and can be assigned to seven components: subjective sleep quality, sleep latency, sleep duration, sleep efficiency, sleep disturbance, use of sleep medication, and daytime dysfunction. These seven categories are assigned scores from 0 to 3, and a higher total score (0 to 21) indicates a worse sleep quality.

##### Saliva and Blood Samples

Saliva samples were collected to measure cortisol, a stress hormone, at three points for each test, before Uchida–Kraepelin stress loading, after Uchida–Kraepelin stress loading, and 60 min after Uchida–Kraepelin stress loading, before intake and at 4, 8, and 12 weeks after starting intake. A blood test was also performed at these times to confirm the safety of ingesting the test substance before intake and at 12 weeks.

#### 2.2.3. Statistical Analyses

The baseline characteristics of the participants were analyzed using one-way analysis of variance (ANOVA). After accounting for normality, two-way repeated-measures ANOVAs were performed since the autonomic nervous system response indices, salivary cortisol, POMS2, SF-36, OSA, and Uchida–Kraepelin test (correct answer rate) were normally distributed. Sessions (weeks 0, 4, 8, and 12) were within-subject independent variables and the study group (placebo and *Euglena* intake groups) were between-group independent variables. If a significant main effect of group, or a session x group, the interaction was found, we then followed up with post hoc comparisons using Dunnett’s test. Kruskal–Wallis test was performed for VAS and PSQI since no normal distribution was found. If the Kruskal–Wallis test was significant, the Steel’s test was then performed. Dunnett’s test or Steel’s test was used to compare the measured values at each measurement time point and/or the degree of change from week 0 to after test among the four groups. Numerical values are shown as means ± standard deviation (SD). Significance levels were set as follows: <5% in two-sided tests indicated a significant difference. In addition, < 10% in the two-tailed test showed a significant trend. R version 3.5.1 (R development Core Team), and JMP (ver. 12) were used for statistical analyses.

## 3. Results

### 3.1. Subjects

**Figure 1:**
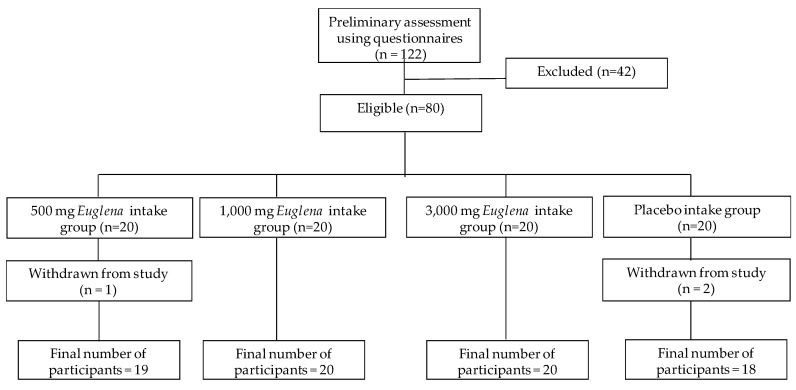
Flow chart for subject participation.

In total, 122 participants were enrolled as the analysis set. After screening, 80 individuals were selected for the study. As three subjects withdrew during the trial, data for 77 subjects were analyzed (Figure 1).

Baseline data for subjects are shown in Table 1. There were no significant differences in parameters measured at baseline between the placebo and *Euglena* intake groups.

### 3.2. Safety Evaluation

Four complaints of symptoms were received during the study period, including fatigue, constipation, headache, and stiffness in the shoulder, although the symptoms were present before study participation and, although the cause could not be identified, the symptoms were mild and thus were judged by the investigator to be unproblematic. No abnormal values were found for vital signs such as blood pressure and pulse, nor in the blood test results. Subjects’ blood pressure, pulse rate, and blood test results are summarized in Supplementary Tables S1 and S2. Although some items significantly differed between weeks, most average values remained within the range of the reference value. No adverse events or other associated issues were observed.

### 3.3. Efficacy Evaluation

#### 3.3.1. Autonomic System Response Indicator Measurements

The low-frequency/high-frequency (LF/HF) ratio is summarized in Table 2. LF and HF, which represent the balance of the sympathetic nervous system and the parasympathetic nervous system in the autonomic nervous system, were measured at three points for each test (before Uchida–Kraepelin stress loading, after Uchida–Kraepelin stress loading, and 60 min after Uchida–Kraepelin stress loading) before intake and at 4, 8, and 12 weeks after starting intake. The LF/HF ratio was 3.7 in the placebo intake group, 3.3 in the *Euglena* 500 mg intake group, 4.1 in the 1000 mg intake group, and 4.7 in the *Euglena* 3000 mg intake group after Uchida–Kraepelin stress loading at week 0. The LF/HF ratio was 6.3 in the placebo intake group, 4.7 in the *Euglena* 500 mg intake group, 2.9 in the 1000 mg intake group, and 5.1 in the *Euglena* 3000 mg intake group after Uchida–Kraepelin stress loading at week 4 of intake. These findings suggest that the continuous intake of *Euglena* reduced disruptions in the balance of the autonomic nervous system. However, there were no significant differences.

The mean change amount of the LF/HF ratio before and after Uchida–Kraepelin loading at week 0 was 0.7. Thus, we defined subjects with a change amount greater than 0.7 as being more prone to workload stress based on Uchida–Kraepelin (Table 3). The LF/HF ratio in the *Euglena* 1000 mg intake group after Uchida–Kraepelin stress loading at week 4 of intake was significantly lower than that in the placebo intake group (*p* = 0.024, Dunnett’s test). This suggests that the intake of *Euglena* 1000 mg regulates the balance of the autonomic nervous system during work stress. No difference was found in the balance of the autonomic nervous system in subjects without Uchida–Kraepelin stress. After week 8 of intake, these differences were difficult to discern.

#### 3.3.2. Analysis of SF-36

Table 4 summarizes the SF-36 results. The role physical (RP) values were significantly higher at weeks 8 and 12 of treatment with *Euglena* 3000 mg intake group (53.5 ± 5.2 and 55.4 ± 3.5) than in the placebo intake group (47.3 ± 8.4 and 49.2 ± 8.2), suggesting that intake of *Euglena* improves RP (*p* = 0.036, *p* = 0.021, Dunnett’s test). At week 12 of intake, vitality (VT) tended to be higher in the 3000 mg *Euglena* intake group (51.2 ± 7.2) than in the placebo group (45.3 ± 5.5), indicating that the continuous intake of *Euglena* may also improve vitality (*p* = 0.065, Dunnett’s test).

#### 3.3.3. POMS Analysis

Table 5 shows the results of a POMS analysis. Results from the intake of 500 mg of *Euglena* indicate that the scores for friendliness were significantly higher than those of the placebo intake group before, immediately after, and 60 min after Uchida–Kraepelin stress loading at week 4 (*p* = 0.009, *p* = 0.007, and *p* = 0.0006, respectively, Dunnett’s test); before, immediately after, and 60 min after Uchida–Kraepelin stress loading at week 8 (*p* = 0.004, *p* = 0.062, and *p* = 0.040, respectively, Dunnett’s test); and immediately after and 60 min after Uchida–Kraepelin stress loading at week 12 (*p* = 0.007, and *p* = 0.003, respectively, Dunnett’s test), respectively.

#### 3.3.4. VAS Analysis

Table 6 and Table 7 show the VAS results. The amount of change in tension from before Uchida–Kraepelin stress loading to 60 min after Uchida–Kraepelin stress loading in week 8 was 0.86 ± 1.67 in the placebo intake group, −0.41 ± 1.61 in the 500 mg *Euglena* intake group, and −0.29 ± 1.43 in the 3000 mg *Euglena* intake group, demonstrating a reduction in tension (*p* = 0.097, *p* = 0.099, Steel’s test). The degree of change in tension from before Uchida–Kraepelin stress loading to 60 min after Uchida–Kraepelin stress loading in week 12 was 0.91 ± 1.77 in the placebo intake group and −0.74 ± 1.51 in the 1000 mg *Euglena* intake group, indicating a significant easing of tension (*p* = 0.023, Steel’s test).

The amount of change in irritability from before Uchida–Kraepelin stress loading to immediately after Uchida–Kraepelin stress loading in week 12 was 1.79 ± 2.00 in the placebo intake group and 0.27 ± 1.59 in the 1000 mg *Euglena* intake group (*p* = 0.057 Steel’s test). The amount of change in irritability from before Uchida–Kraepelin stress loading to 60 min after Uchida–Kraepelin stress loading in week 12 was 1.29 ± 2.02 in the placebo intake group and −0.54 ± 1.30 in the 1000 mg *Euglena* intake group, showing a significant easing of irritability caused by Uchida–Kraepelin stress (*p* = 0.008, Steel’s test).

The amount of change in the level of subjective satisfaction with sleep from week 0 to 4 was -0.89 ± 1.44 in the placebo intake group and −2.61 ± 1.75 in the 3000 mg *Euglena* intake group, showing a significant increase in the level of subjective satisfaction with sleep (*p* = 0.007, Steel’s test). The amount of change in the level of subjective satisfaction with sleep from week 0 to 12 was −0.65 ± 1.22 in the placebo intake group, −2.22 ± 3.08 in the 500 mg *Euglena* intake group, −2.69 ± 2.31 in the 1000 mg *Euglena* intake group, and −3.41 ± 2.26 in the 3000 mg *Euglena* intake group, showing a significant difference increase in the level of subjective satisfaction with sleep (*p* = 0.028, *p* = 0.012, *p* = 0.0006, respectively, Steel’s test).

#### 3.3.5. OSA Analysis

Table 8 shows the OSA results. Sleepiness on rising was significantly higher after 8 weeks of the continuous intake of *Euglena* 500 mg (47.7 ± 9.0) than in the placebo intake group (41.5 ± 5.3) (*p* = 0.027, Dunnett’s test); at week 12 after the continuous intake of *Euglena* 500 mg (48.9 ± 10.8) than in the placebo intake group (41.4 ± 7.2) (*p* = 0.027, Dunnett’s test); and at week 12 after the continuous intake of *Euglena* 3000 mg (48.3 ± 8.9) than in the placebo intake group (41.4 ± 7.2) (*p* = 0.040, Dunnett’s test). These results suggest that the quality of sleep may improve with the intake of *Euglena.*

The “refreshing” index tended to be higher at week 8 after the continuous intake of *Euglena* 500 mg (47.0 ± 8.5) than in the placebo intake group (41.1 ± 4.7) (*p* = 0.055, Dunnett’s test). This index was significantly higher in week 12 after the continuous intake of *Euglena* 500 mg (48.4 ± 8.0) than in the placebo intake group (41.6 ± 7.0) (*p* = 0.032, Dunnett’s test). This index was significantly higher at week 12 after the continuous intake of *Euglena* 3000 mg (49.0 ± 8.2) than in the placebo intake group (41.6 ± 7.0) (*p* = 0.016, Dunnett’s test). These results suggest that the quality of sleep may improve with the intake of *Euglena.*

Sleep length tended to be longer in week 12 after the continuous intake of *Euglena* 500 mg (51.3 ± 9.3) than in the placebo intake group (44.5 ± 7.1) (*p* = 0.060, Dunnett’s test). Sleep length tended to be longer in week 12 after the continuous intake of *Euglena* 3000 mg (50.7 ± 10.9) than in the placebo intake group (44.5 ± 7.1) (*p* = 0.089, Dunnett’s test). These findings suggest that the quality of sleep may improve with the intake of *Euglena*.

#### 3.3.6. PSQI Analysis

Table 9 shows the results of a PSQI analysis. The total PSQI score was significantly lower at week 4 after the continuous intake of *Euglena* 3000 mg (5.1 ± 2.0) than in the placebo intake group (7.1 ± 2.6) (*p* = 0.039, Steel’s test); at week 12 after the continuous intake of *Euglena* 3000 mg (4.2 ± 2.1) than in the placebo intake group (6.3 ± 2.3) (*p* = 0.026, Steel’s test); and after the continuous intake of *Euglena* 1000 mg (4.7 ± 2.1) compared with the placebo intake group (6.3 ± 2.3) (*p* = 0.070, Steel’s test). These results suggest that the quality of sleep may improve with the intake of *Euglena.*

#### 3.3.7. Analysis of Saliva Samples

No significant differences were observed in the salivary cortisol levels.

#### 3.3.8. Uchida–Kraepelin Results

Table 10 shows the correct answer rates in the Uchida–Kraepelin test. The correct answer rate in the first half increased significantly in weeks 8 and 12 of intake of *Euglena* 1000 mg (*p* = 0.009, and *p* = 0.020, respectively, Dunnett’s test).

The correct answer rate in the first half tended to increase in week 8 with the continuous intake of *Euglena* 500 mg (*p* = 0.083, Dunnett’s test), in week 12 with the continuous intake of *Euglena* 3000 mg (*p* = 0.067, Dunnett’s test).

## 4. Discussion

We conducted a double-blind placebo-controlled trial to evaluate the effects of *Euglena* intake on various properties, such as the autonomic nervous system, psychological parameters, and quality of sleep. Our results indicated that the intake of *Euglena* regulates the autonomic nervous system balance and improves various symptoms caused by stress loading. In addition, we observed no adverse events with a maximum intake of 3000 mg of *Euglena* per day, confirming that this dose is safe.

The subjects included in the study were concerned about a lack of motivation and a decline in the quality of sleep. However, the sympathetic nervous system was in a dominant state in these individuals, with a high LF/HF ratio, which is an indicator of the autonomic nervous system balance, exceeding the normal value of 2.0. Previous studies have shown that Uchida–Kraepelin stress affects heart rate variability [31,32,33,34]. We selected subjects who felt that taking the Uchida–Kraepelin test was stressful had a substantial increase in their LF/HF ratios. We found that the intake of *Euglena* at 1000 mg suppressed the LF/HF ratio compared with that in the placebo intake group. When the LF/HF ratio was broken down into the components LF and HF, a decrease in LF had a relatively large impact upon 500 mg and 1000 mg Euglena intake. Therefore, *Euglena* may have suppressed the excitation of the sympathetic nervous system. The intake of *Euglena* significantly suppressed the LF/HF ratios in individuals subjected to work stress and appeared to regulate the autonomic nervous system balance. The most effective dose was 1000 mg.

Irritability and tension during work stress were maintained at high levels even 60 min after exposure to work stress in the placebo intake group; however, these parameters were suppressed in the *Euglena* intake groups of 500 mg or more and were significantly suppressed in the 1000 mg intake group, indicating an improvement in the psychological state. In other words, the intake of *Euglena* may have alleviated the tension caused by Uchida–Kraepelin stress loading.

In a POMS2 analysis, which evaluates emotions, the score for friendship (F) was significantly higher in the group taking 500 mg of *Euglena* per day at weeks 4, 8, and 12 after starting intake, both before and after Uchida–Kraepelin stress loading. This suggests that the continuous intake of 500 mg *Euglena* can enhance friendliness. However, this did not occur upon taking *Euglena* at doses other than 500 mg. In SF-36, which evaluates health-related quality of life, a significant difference was observed in daily physical function (role physical), which represents physical fatigue.

Improvement of sleep quality with the intake of *Euglena* was confirmed based on three sleep tests: VAS, OSA, and PSQI. These effects increased in a dose- and time-dependent manners. The parasympathetic nervous system is enhanced at the onset of sleep [35]; accordingly, the improvement in sleep quality by *Euglena* intake was likely due to an improvement in the balance of the autonomic nervous system.

*Euglena* intake also increased the correct answer rate on the Uchida–Kraepelin test. These findings suggest that the continuous intake of *Euglena* may improve concentration and work efficiency.

The autonomic nervous system includes the sympathetic nervous system and the parasympathetic nervous system. It regulates various functions, such as respiration/circulation, digestion/absorption, secretion, and metabolism, and serves an important role in the maintenance of homeostasis in the body. However, when stress signals are transmitted to the hypothalamus in the brain, the autonomic nervous system is excited, depending on the type of stress. An imbalance in the autonomic nervous system due to excessive stress may prevent the proper activation of the sympathetic or parasympathetic nervous system. These conditions may cause excessive irritability, an inability to concentrate, deterioration of sleep quality, weakened immunity, and various ailments, including problems with bowel movements. For example, patients with chronic fatigue syndrome caused by stress experience a decrease in parasympathetic nervous system function and a relative increase in sympathetic nervous system function, depending on the degree of subjective fatigue evaluated by the VAS. This shows that there is a relationship between fatigue and the autonomic nervous system [36]. Therefore, it is important to maintain a well-balanced autonomic nervous system. In this study, the intake of *Euglena* improved the autonomic nervous system balance, thereby improving irritability and tension as well as the quality of daily sleep. Previous studies have evaluated improvements in autonomic nervous system activity after nutritional supplementation in healthy subjects [37,38,39,40]. When stress is transmitted to the hypothalamus in the brain, a signal is sent to excite the autonomic nervous system, depending on the type of stress, and a command is simultaneously sent to the pituitary gland to stimulate the thyroid, adrenal medulla, and adrenal cortex via the release of hormones. In addition, signals from the hypothalamus stimulate the adrenal cortex in response to stress; the pituitary gland secretes the adrenocorticotrophic hormone, adrenocorticotropic hormone (ACTH), and the adrenal cortex secretes the hormone cortisol. In this way, the hypothalamus, which controls the autonomic nervous system, also controls the pituitary gland, which secretes various hormones and neurotransmitters. Therefore, a mutual relationship exists whereby an imbalance in the autonomic nervous system affects the secretion of hormones and neurotransmitters, thereby affecting the balance of the autonomic nervous system. Proteins, B vitamins, and minerals, such as magnesium, are required for hormones and neurotransmitters. A lack of vitamin B6 makes it difficult for the body to produce serotonin and melatonin, leading to insomnia and autonomic nervous system ataxia. The overconsumption of zinc is related to stress, and a shortage of vitamin B makes it difficult to produce energy in the mitochondria, which increases fatigue and malaise. *Euglena* consist of a wide variety of nutrients, such as vitamins, minerals, amino acids, and unsaturated fatty acids. The ingestion of *Euglena* assists in replenishing these nutrients in the body, which not only improves autonomic nervous system activity but also normalizes the secretion of hormones and neurotransmitters, contributing to the regulation of the autonomic nervous system balance. In this study, we found no changes in cortisol levels, which suggests that hormone levels are being affected rather than cortisol levels and that the stress of Uchida–Kraepelin may not affect cortisol production [41]; this warrants further investigation.

Meanwhile, previous studies have used in vitro cell-type-specific calcium imaging to prove that *Euglena* directly induces Ca^2+^ signaling in dorsal root ganglia (DRG) neurons, suggesting that *Euglena* excites visceral afferents [42]. Although the mechanism underlying the beneficial effects of *Euglena* in our study remains unclear, its ability to excite neurons may contribute to improvements in health-related quality of life [12]. Further studies are needed to better clarify the physiological role of visceral afferents in response to food constituents.

The storage polysaccharide paramylon in *Euglena* regulates the immune balance. Previous studies of mouse models have confirmed that the ingestion of *Euglena* or paramylon alleviates the symptoms of influenza infection [11], rheumatoid arthritis [43], and atopic dermatitis [44]. The autonomic nervous system is involved in the regulation of immunity, and immunity is generally enhanced in situations where the parasympathetic nervous system is dominant, contributing to defense against infection [16,17]. Cortisol also suppresses the immune system [45]. Previous studies have investigated the ability of paramylon to regulate immunity by binding to Dectin-1 expressed on intestinal immune cells [46,47]; however, it could also mediate immunity by the regulation of the autonomic nervous system and endocrine system.

The results of this study suggest that the intake of *Euglena*-containing food may regulate the autonomic nervous system balance under stress and may have favorable effects on work efficiency, psychological factors, and sleep quality. However, this study had some limitations. First, although this study was conducted with a small homogeneous cohort of individuals from one population, future studies are required to further elucidate these aspects with various ethnicities and people with different backgrounds and stress levels. Furthermore, although some of the effects were dependent on intake dose, other effects were observed for which an optimal dose may exist independent of intake, thus requiring further investigation. In the future, we would like to further investigate the mechanism underlying these beneficial effects as well.

## Figures and Tables

**Table 1:** Baseline data for subjects. After accounting for normality, the baseline characteristics of the participants were analyzed by one-way ANOVA or Kruskal–Wallis test.

									SF-36	LH/HF			PSQI
Group	*n*	Male (*n*)	Female (*n*)		Age (Years)	Height	Weight	BMI	VT	Before	After	Δ After	Total Score
Placebo	18	8	10	Mean	47.3	164.2	58.9	21.7	35.8	3.1	3.7	0.5	7.7
				SD	9.5	8.0	8.5	1.7	12.1	2.5	3.5	2.9	2.6
*Euglena*	19	9	10	Mean	47.8	165.2	60.1	22.0	39.8	3.1	3.3	0.3	8.1
500 mg				SD	11.5	6.4	9.8	2.8	14.6	2.2	2.5	2.2	3.1
*Euglena*	20	9	11	Mean	46.0	164.6	59.9	22.0	33.2	3.1	4.1	1.0	7.4
1000 mg				SD	10.7	10.8	10.9	2.3	12.4	2.4	6.6	5.8	2.4
*Euglena*	20	9	11	Mean	48.0	162.8	59.9	22.5	34.1	3.9	4.7	0.8	6.8
3000 mg				SD	9.7	8.4	10.6	2.8	11.7	5.0	3.5	3.8	2.2
*p*-value (one-way ANOVA)					0.931	0.838	0.980	0.804	0.381	0.822	0.775	0.931	-
*p*-value (Kruskal–Wallis test)					-	-	-	-	-	-	-	-	0.556

BMI: body mass index, SF-36: 36-Item Short-Form Health Survey, VT: vitality, LF/HF: low-frequency/high-frequency, PSQI: Pittsburgh Sleep Quality Index.

**Table 2:** Summary of LF/HF ratios, an indicator of autonomic nervous system balance. Low frequency (LF) and high frequency (HF) indicate sympathetic and parasympathetic nervous, respectively. After accounting for normality, a two-way repeated measure ANOVA was performed. The results were not significantly different.

			LF/HF 0 Weeks			LF/HF 4 Weeks			LF/HF 8 Weeks			LF/HF 12 Weeks		
Group	*n*		Before	After	60 min after	Before	After	60 min after	Before	After	60 min after	Before	After	60 min after
Placebo	18	Mean	3.1	3.7	2.7	4.0	6.3	4.8	3.7	4.3	4.3	3.1	3.3	2.7
		SD	2.5	3.5	2.6	5.2	6.3	6.8	3.7	3.4	3.3	2.3	2.7	2.6
*Euglena*	19	Mean	3.1	3.3	2.8	4.4	4.7	3.9	3.2	3.4	2.5	3.6	3.9	2.9
500 mg		SD	2.2	2.5	2.0	5.2	4.3	4.1	3.3	2.8	1.9	3.0	3.4	2.7
*Euglena*	20	Mean	3.1	4.1	3.4	3.5	2.9	2.1	2.6	3.3	3.1	2.5	3.1	2.7
1000 mg		SD	2.4	6.6	5.2	3.3	2.2	1.2	3.3	2.7	4.1	2.7	2.4	2.8
*Euglena*	20	Mean	3.9	4.7	4.5	3.7	5.1	3.5	3.7	3.9	3.9	4.0	4.1	2.7
3000 mg		SD	5.0	3.5	5.3	3.7	5.3	2.7	3.2	3.2	4.5	3.4	2.9	1.8

LF/HF: low-frequency/high-frequency.

**Table 3:** Summary of LF/HF ratios, an indicator of autonomic nervous system balance (Subjects defined as susceptible to stress). After accounting for normality, a two-way repeated measure ANOVA was performed. The results showed a session x group interaction in “After” (*p* = 0.036) and were followed up with a post-hoc comparison using Dunnett’s test. Dunnett’s test was used for intergroup comparisons of the means of the placebo group and *Euglena* intake groups before and after Uchida–Kraepelin stress loading, and 60 min after loading at 0, 4, 8, and 12 weeks.

			LF/HF 0 Weeks			LF/HF 4 Weeks			LF/HF 8 Weeks			LF/HF 12 Weeks		
Group	*n*		Before	After	60 min after	Before	After	60 min after	Before	After	60 min after	Before	After	60 min after
Placebo	8	Mean	3.1	6.0	4.2	4.0	10.3	7.0	5.4	5.4	5.5	3.1	4.2	3.2
		SD	2.5	4.1	3.3	3.4	7.8	9.7	4.9	4.3	3.1	2.9	3.6	3.8
*Euglena*	8	Mean	2.5	4.6	2.7	4.5	5.0	4.4	2.7	4.9	2.3	3.4	4.0	3.5
500 mg		SD	1.8	2.4	2.0	5.2	4.8	4.8	3.3	3.5	1.1	2.6	3.3	3.1
*Euglena*	8	Mean	3.1	8.1	5.9	4.8	2.6 *	2.6	3.8	4.9	5.7	2.8	2.5	3.5
1000 mg		SD	2.1	9.2	7.8	4.4	1.1	1.2	4.7	3.4	5.6	3.3	1.6	3.9
*Euglena*	10	Mean	3.3	6.5	6.6	5.2	7.4	4.9	4.7	5.4	5.9	3.8	4.6	3.3
3000 mg		SD	2.3	3.1	6.2	4.6	6.2	3.1	3.5	3.7	5.7	2.4	2.8	2.0

* *p* < 0.05 vs. the placebo intake group. LF/HF: low-frequency/high-frequency.

**Table 4:** Analyses of role physical (RP) and vitality (VT) based on SF-36. After accounting for normality, a two-way repeated measure ANOVA was performed. The results showed a main effect of group in RP (*p* = 0.023) and a session x group interaction in VT (*p* = 0.052). Therefore, we followed up with post-hoc comparisons using Dunnett’s test. Dunnett’s test was used for intergroup comparisons of mean values in the placebo intake group and *Euglena* intake group at 0, 4, 8, and 12 weeks.

	Group	*n*		0 Weeks	4 Weeks	8 Weeks	12 Weeks
RP	Placebo	18	Mean	44.2	48.5	47.3	49.2
			SD	10.0	8.7	8.4	8.2
	*Euglena*	19	Mean	43.9	48.7	48.7	50.2
	500 mg		SD	10.3	8.8	6.7	7.8
	*Euglena*	20	Mean	44.3	44.1	47.5	50.2
	1000 mg		SD	9.6	10.4	9.5	7.6
	*Euglena*	20	Mean	46.9	54.1	53.5 *	55.4 *
	3000 mg		SD	8.3	3.9	5.2	3.5
VT	Placebo	18	Mean	39.9	46.8	44.3	45.3
			SD	5.9	6.5	8.7	5.4
	*Euglena*	19	Mean	41.8	46.2	47.8	49.0
	500 mg		SD	7.1	9.4	10.9	9.4
	*Euglena*	20	Mean	38.6	42.7	43.9	45.1
	1000 mg		SD	6.0	8.7	10.1	9.1
	*Euglena*	20	Mean	39.0	48.6	49.7	51.2 ^†^
	3000 mg		SD	5.7	7.3	6.2	7.2

* *p* < 0.05, ^†^ *p* < 0.1 vs. the placebo intake group. SF-36: 36-Item Short-Form Health Survey, RP: role physical, VT: vitality.

**Table 5:** Analyses of friendliness as determined by POMS2. After accounting for normality, a two-way repeated measure ANOVA was performed. The results showed a session x group interaction in “Before,” “After,” and “60 min after” (*p* = 0.075, *p* = 0.009, and *p* = 0.012, respectively) and a main effect of group in “Before,” “After,” and “60 min after” (*p* = 0.058, *p* = 0.055, and *p* = 0.018, respectively). Therefore, we followed up with post-hoc comparisons using Dunnett’s test. Dunnett’s test was used for intergroup comparisons of the mean values in the placebo intake group and *Euglena* intake groups before and after Uchida-Kraepelin stress loading and 60 min after loading at 0, 4, 8, and 12 weeks.

			0 Weeks			4 Weeks			8 Weeks			12 Weeks		
Group	*n*		Before	After	60 min after	Before	After	60 min after	Before	After	60 min after	Before	After	60 min after
Placebo	18	Mean	44.6	41.4	39.3	44.0	39.3	36.9	41.1	39.4	39.2	44.1	37.7	37.2
		SD	11.4	9.9	10.9	9.6	10.3	9.5	11.1	11.1	11.0	10.1	9.7	8.9
*Euglena*	19	Mean	48.5	44.4	45.3	54.2 **	51.8 **	52.5 ***	52.9 **	48.2 ^†^	48.9 *	50.8	49.7 **	50.6 **
500 mg		SD	11.7	11.1	13.3	12.1	13.0	13.6	13.0	14.5	15.0	12.9	15.1	15.9
*Euglena*	20	Mean	45.7	41.4	41.9	45.5	42.3	42.4	45.6	40.8	40.3	44.9	42.1	41.0
1000 mg		SD	8.0	9.3	8.6	8.9	11.9	10.3	6.1	8.1	8.2	8.3	9.1	9.8
*Euglena*	20	Mean	48.1	44.3	43.3	46.9	42.4	42.9	47.6	43.5	41.9	44.9	42.9	41.8
3000 mg		SD	10.4	11.0	12.2	9.8	13.2	14.3	11.7	11.6	12.1	12.5	12.0	11.8

*** *p* < 0.001, ** *p* < 0.01, * *p* < 0.05, ^†^ *p* < 0.1 vs. the placebo group. POMS2: Profile of Mood States 2nd Edition.

**Table 6:** Analyses of tension and irritability measured by the VAS. After accounting for normality, Kruskal–Wallis test was performed. If the Kruskal–Wallis test was significant, the Steel’s test was then performed. Steel’s test was used for intergroup comparison of the mean change from before Uchida–Kraepelin stress loading to immediately after, and 60 min after stress loading at 0, 4, 8, and 12 weeks in the placebo and *Euglena* intake groups.

				0 Weeks			4 Weeks			8 Weeks			12 Weeks		
	Group	*n*		Before	After	60 min after	Before	After	60 min after	Before	After	60 min after	Before	After	60 min after
Tension	Placebo	18	Mean	5.14	4.84	4.82	4.21	4.29	4.18	3.88	4.77	4.74	4.11	5.58	5.02
			SD	1.54	2.26	2.33	1.80	2.09	2.10	1.61	1.92	1.94	1.42	1.46	1.89
			Mean (Amount of change)	-	−0.30	−0.33	-	0.09	−0.02	-	0.89	0.86	-	1.47	0.91
			SD (Amount of change)	-	1.78	1.66	-	1.55	1.99	-	1.47	1.67	-	1.86	1.77
	*Euglena*	19	Mean	3.74	3.39	2.68 *	3.30	3.09	2.77	2.63	2.59 **	2.22 **	2.81	3.32 *	2.71 **
	500 mg		SD	1.79	2.12	1.80	2.27	2.36	1.96	1.95	1.85	1.77	1.95	2.16	2.11
			Mean (Amount of change)	-	−0.35	−1.05	-	−0.21	−0.53	-	−0.04	−0.41 ^^†^^	-	0.51	−0.09
			SD (Amount of change)	-	1.66	1.94	-	1.56	1.31	-	1.72	1.61	-	1.67	1.94
	*Euglena*	20	Mean	4.84	4.58	3.84	3.74	4.21	3.56	3.20	3.92	3.33 ^†^	3.94	3.69 *	3.20 *
	1000 mg		SD	1.50	1.89	1.78	1.51	1.41	1.49	1.50	1.95	1.70	1.65	1.81	1.65
			Mean (Amount of change)	-	−0.26	−1.00	-	0.47	−0.18	-	0.72	0.13	-	−0.25	−0.74 *
			SD (Amount of change)	-	1.96	1.87	-	1.65	1.38	-	1.29	1.08	-	1.92	1.52
	*Euglena*	20	Mean	4.61	4.29	3.59	3.23	3.60	2.82 ^†^	3.06	3.37 ^†^	2.78 **	3.15	3.38 **	2.72 **
	3000 mg		SD	1.33	2.21	2.42	1.66	1.89	1.75	1.85	1.90	1.54	1.81	2.14	2.17
			Mean (Amount of change)	-	−0.32	−1.02	-	0.37	−0.41	-	0.31	−0.29 ^†^	-	0.23	−0.43
			SD (Amount of change)	-	1.42	2.13	-	2.08	2.50	-	1.57	1.43	-	2.12	2.14
*p*-value (Kruskal–Wallis test)				0.0663 ^†^	0.1442	0.032 *	0.4204	0.2222	0.0975 ^†^	0.1562	0.0102 *	0.0011 **	0.0988 ^†^	0.0049 **	0.0038 **
*p*-value (Kruskal–Wallis test/Amount of change)					0.9468	0.6333		0.4346	0.6664		0.1564	0.0906 ^†^		0.14	0.0682 ^†^
Irritability	Placebo	18	Mean	6.09	5.04	5.41	4.64	4.62	4.63	4.23	5.33	5.32	4.08	5.88	5.38
			SD	1.49	2.64	2.49	2.33	2.36	2.33	2.02	1.93	1.89	1.57	1.62	2.09
			Mean (Amount of change)	-	−1.06	−0.69	-	−0.02	−0.01	-	1.11	1.09	-	1.79	1.29
			SD (Amount of change)	-	2.99	2.91	-	2.67	2.39	-	1.99	1.92	-	2.00	2.02
	*Euglena*	19	Mean	4.60	3.36	3.23 ^†^	2.85 ^†^	3.05	2.72 *	2.61	2.97 *	2.29 **	2.56 ^†^	3.33 *	2.97 *
	500 mg		SD	2.45	2.56	3.02	2.50	2.56	2.46	2.15	2.56	2.30	2.03	2.87	2.55
			Mean (Amount of change)	-	−1.24	−1.37	-	0.21	−0.13	-	0.36	−0.32	-	0.76	0.41
			SD (Amount of change)	-	3.13	3.49	-	2.46	2.08	-	2.19	2.25	-	1.78	1.99
	*Euglena*	20	Mean	5.54	5.70	4.69	3.61	5.04	4.82	3.58	4.65	4.11	4.21	4.48	3.67 ^†^
	1000 mg		SD	1.97	2.52	2.52	2.42	2.53	2.63	2.37	2.52	2.51	2.49	2.65	2.40
			Mean (Amount of change)	-	0.16	−0.86	-	1.43	1.21	-	1.07	0.54	-	0.27 ^†^	−0.54 **
			SD (Amount of change)	-	1.68	1.53	-	2.15	2.39	-	2.16	2.07	-	1.59	1.30
	*Euglena*	20	Mean	4.67	4.49	3.62 ^†^	3.21	4.03	3.25	3.44	4.11	3.12 **	2.83 ^†^	4.35	3.42 *
	3000 mg		SD	2.68	2.53	2.46	2.25	2.40	2.02	2.75	2.47	1.98	2.14	2.45	2.45
			Mean (Amount of change)	-	−0.18	−1.05	-	0.82	0.04	-	0.68	−0.32	-	1.52	0.59
			SD (Amount of change)	-	3.27	2.61	-	2.62	2.47	-	2.92	2.34	-	2.87	2.67
*p*-value (Kruskal–Wallis test)				0.2245	0.0641 ^†^	0.0649 ^†^	0.1172	0.0827 ^†^	0.0155 *	0.2593	0.0335 *	0.0007 ***	0.0317 *	0.0352 *	0.0199 *
*p*-value (Kruskal–Wallis test/Amount of change)				-	0.3628	0.8083	-	0.3075	0.3036	-	0.6082	0.3015	-	0.0927 ^†^	0.0323 *

*** *p* < 0.001, ** *p* < 0.01, * *p* < 0.05, ^†^ *p* < 0.1 vs. the placebo intake group. VAS: visual Analog Scale.

**Table 7:** Analyses of the level of subjective satisfaction with sleep measured by the VAS. After accounting for normality, Kruskal–Wallis test was performed. If the Kruskal–Wallis test was significant, the Steel’s test was then performed. Steel’s test was used for intergroup comparison of the mean change at 0, 4, 8, and 12 weeks in the placebo and *Euglena* intake groups.

	Group	*n*		0 Weeks	4 Weeks	8 Weeks	12 Weeks	Δ 4 Weeks	Δ 8 Weeks	Δ 12 Weeks
Sleep	Placebo	18	Mean	6.93	6.04	5.84	6.28	−0.89	−1.09	−0.65
			SD	2.02	2.38	2.22	1.95	1.44	1.51	1.22
	*Euglena*	19	Mean	6.77	5.73	4.74	4.55 ^†^	−1.04	−1.87	−2.22 *
	500 mg		SD	2.49	2.89	2.62	2.66	1.43	1.69	3.08
	*Euglena*	20	Mean	7.35	5.50	5.47	4.66 ^†^	−1.85	−1.88	−2.69 *
	1000 mg		SD	1.19	2.34	2.22	2.47	1.85	2.29	2.31
	*Euglena*	20	Mean	7.65	5.04	4.98	4.24 *	−2.61 **	−2.67	−3.41 ***
	3000 mg		SD	1.50	2.52	2.50	2.37	1.75	1.93	2.26
*p*-value (Kruskal–Wallis test)				0.6878	0.5877	0.5464	0.0492 *	0.0040 **	0.1027	0.0014 **

*** *p* < 0.001, ** *p* < 0.01, * *p* < 0.05, ^†^ *p* < 0.1 vs. the placebo intake group. VAS: visual Analog Scale.

**Table 8:** Summary of OSA analysis on Sleepiness on rising, Refreshing, and Sleep length. After accounting for normality, a two-way repeated measure ANOVA was performed. The results showed a session x group interaction (*p* = 0.054, *p* = 0.016, *p* = 0.003) and a main effect of group (*p* = 0.052, *p* = 0.089, *p* = 0.780), therefore, we followed up with post-hoc comparisons using Dunnett’s test. Dunnett’s test was used for intergroup comparisons of mean values in the placebo group and *Euglena* intake group at 0, 4, 8, and 12 weeks.

	Group	*n*		0 Weeks	4 Weeks	8 Weeks	12 Weeks	Δ 4 Weeks	Δ 8 Weeks	Δ 12 Weeks
Sleepiness on rising	Placebo	18	Mean	41.8	40.6	41.5	41.4	−1.2	−0.3	−0.4
			SD	7.0	5.7	5.3	7.2	6.0	3.4	5.9
	*Euglena*	19	Mean	42.1	43.4	47.7 *	48.9 *	1.3	5.3 *	6.7 *
	500 mg		SD	9.1	6.9	9.0	10.8	6.6	7.2	8.6
	*Euglena*	20	Mean	38.1	41.2	43.6	43.5	3.1	5.5 *	5.4 ^†^
	1000 mg		SD	3.3	5.7	6.0	6.5	5.7	7.4	8.2
	*Euglena*	20	Mean	42.7	44.5	44.7	48.3 *	1.9	2.1	5.7 ^†^
	3000 mg		SD	6.5	6.2	7.5	8.9	7.0	7.9	10.2
Refreshing	Placebo	18	Mean	41.2	41.5	41.1	41.6	0.3	−0.2	0.3
			SD	7.6	6.7	4.7	7.0	5.8	5.6	6.4
	*Euglena*	19	Mean	43.2	42.0	47.0 ^†^	48.4 *	−1.2	3.6	5.2
	500 mg		SD	5.7	9.0	8.5	8.0	9.2	9.4	9.7
	*Euglena*	20	Mean	37.3	42.0	43.9	44.1	4.8	6.7 *	6.8 ^†^
	1000 mg		SD	5.4	7.8	7.6	8.9	7.9	10.0	9.6
	*Euglena*	20	Mean	38.9	44.5	45.4	49.0 *	5.5	6.5 ^†^	10.1 *
	3000 mg		SD	6.9	6.6	8.5	8.2	8.9	8.4	8.8
Sleep length	Placebo	18	Mean	43.5	47.7	44.1	44.5	4.2	0.6	1.0
			SD	8.2	8.4	8.9	7.1	7.0	5.8	6.5
	*Euglena*	19	Mean	46.4	42.5	46.8	51.3 ^†^	−3.8 *	0.2	4.9
	500 mg		SD	6.9	13.9	8.0	9.3	12.2	7.3	9.9
	*Euglena*	20	Mean	42.7	43.9	47.2	46.3	1.2	4.4	3.6
	1000 mg		SD	6.4	8.1	10.9	8.1	8.3	11.2	9.5
	*Euglena*	20	Mean	40.8	45.2	49.1	50.7 ^†^	4.4	8.3 *	9.9 **
	3000 mg		SD	9.4	6.9	10.0	10.9	8.0	10.7	7.7

** *p* < 0.01, * *p* < 0.05, ^†^ *p* < 0.1 vs. the placebo intake group. OSA: Ogri-Shirakawa-Azumi Sleep Inventory.

**Table 9:** Analysis of sleep based on PSQI. After accounting for normality, Kruskal–Wallis test was performed. If the Kruskal–Wallis test was significant, the Steel’s test was then performed. Steel’s test was used for intergroup comparisons of mean values in the placebo group and *Euglena* intake groups at 0, 4, 8, and 12 weeks.

	Group	*n*		0 Weeks	4 Weeks	8 Weeks	12 Weeks
Total score	Placebo	18	Mean	7.7	7.1	6.2	6.3
			SD	2.6	2.6	2.4	2.3
	*Euglena*	19	Mean	8.1	5.9	5.5	5.7
	500 mg		SD	3.1	2.2	1.5	2.3
	*Euglena*	20	Mean	7.4	5.8	5.4	4.7 ^†^
	1000 mg		SD	2.4	2.3	2.6	2.1
	*Euglena*	20	Mean	6.8	5.1 *	4.9	4.2 *
	3000 mg		SD	2.2	2.0	2.4	2.1
*p*-value (Kruskal–Wallis test)				0.5557	0.0803 ^†^	0.2304	0.0243 *

* *p* < 0.05, ^†^ *p* < 0.1 vs. the placebo intake group. PSQI: Pittsburgh Sleep Quality Index.

**Table 10:** Correct answer rates (%) for the Uchida–Kraepelin test. After accounting for normality, a two-way repeated measure ANOVA was performed. The results showed a main effect of group (*p* = 0.098) in “First half;” therefore, we followed up with post-hoc comparisons using Dunnett’s test. Dunnett’s test was used for intergroup comparisons of mean values in the placebo group and *Euglena* intake groups at 0, 4, 8, and 12 weeks.

			0 Weeks		4 Weeks		8 Weeks		12 Weeks	
Group	*n*		First Half	Latter Half	First Half	Latter Half	First Half	Latter Half	First Half	Latter Half
Placebo	18	Mean	98.7	98.8	98.9	98.7	98.7	99.0	98.6	98.7
		SD	1.4	1.2	1.3	1.2	1.4	1.0	1.4	1.2
*Euglena*	19	Mean	98.7	99.1	99.1	99.2	99.3 ^†^	99.2	99.3	99.2
500 mg		SD	1.5	0.9	0.9	0.8	0.8	1.0	1.0	1.1
*Euglena*	20	Mean	99.2	99.2	99.3	99.2	99.6 **	99.5	99.5 *	99.4
1000 mg		SD	0.5	0.5	0.7	0.6	0.5	0.5	0.6	0.6
*Euglena*	20	Mean	99.1	99.0	99.0	99.2	99.2	99.2	99.4 ^†^	99.4
3000 mg		SD	0.7	1.2	0.8	0.7	0.6	0.7	0.8	0.5

** *p* < 0.01, * *p* < 0.05, ^†^ *p* < 0.1 vs. the placebo intake group.

## Supplementary Materials

Supplementary materials can be found at https://www.mdpi.com/2072-6643/12/11/3243/s1, Table S1: Summary of blood test results. Table S2: Summary of blood pressure and pulse rate test results.
