# Supplementary material for: Effects of Euglena gracilis Intake on Mood and Autonomic Activity under Mental Workload, and Subjective Sleep Quality: A Randomized, Double-Blind, Placebo-Controlled Trial
Source: Nutrients. 2020 Oct 23;12(11):3243. doi: 10.3390/nu12113243 (PMC7690740; doi:10.3390/nu12113243)
Supplement: Supplementary file 1 [file nutrients-12-03243-s001.pdf]

Supplemental Table 1: Summary of blood test results. Weeks 0 and 12 measurements and significant differences with corresponding paired t-tests within each group are shown. Although some items significantly differed between weeks, most averages of each value were within the range of the reference value. The investigators found no problems.

Parameter	Group	n		0 weeks	12 weeks	Between Study Day *p* -Value	Reference Value
Serum Total Protein (g/dL)	Placebo	18	Mean	7.1	7.1	0.848	6.7 - 8.3
			SD	0.4	0.4		
	Euglena 500 mg	19	Mean	7.3	7.5	0.198	
			SD	0.4	0.5		
	Euglena 1,000 mg	20	Mean	7.3	7.3	0.638	
			SD	0.4	0.4		
	Euglena 3,000 mg	20	Mean	7.2	7.2	0.903	
			SD	0.3	0.2		
Alb(Albumin) (g/dL)	Placebo	18	Mean	4.5	4.4	0.255	3.8 - 5.2
			SD	0.3	0.4		
	Euglena 500 mg	19	Mean	4.5	4.6	0.513	
			SD	0.3	0.3		
	Euglena 1,000 mg	20	Mean	4.5	4.5	0.304	
			SD	0.3	0.3		
	Euglena 3,000 mg	20	Mean	4.5	4.4	0.081	
			SD	0.3	0.2		
Total Bilirubin (mg/dL)	Placebo	18	Mean	1.0	0.8	0.018	0.2 - 1.2
			SD	0.4	0.3		
	Euglena 500 mg	19	Mean	0.9	0.8	0.206	
			SD	0.3	0.3		
	Euglena 1,000 mg	20	Mean	0.9	0.9	0.933	
			SD	0.3	0.3		
	Euglena 3,000 mg	20	Mean	0.9	0.7	0.035	
			SD	0.4	0.3		
AST(GOT) (U/L)	Placebo	18	Mean	19.2	19.2	0.936	10 - 40
			SD	4.8	5.5		
	Euglena 500 mg	19	Mean	19.1	19.5	0.591	
			SD	5.0	4.4		
	Euglena 1,000 mg	20	Mean	21.3	23.4	0.102	
			SD	7.2	8.7		
	Euglena 3,000 mg	20	Mean	19.9	22.0	0.035	
			SD	5.5	7.2		
ALT(GPT) (U/L)	Placebo	18	Mean	15.7	16.4	0.502	5 - 45
			SD	7.3	8.5		
	Euglena 500 mg	19	Mean	15.9	14.9	0.318	
			SD	7.2	5.2		
	Euglena 1,000 mg	20	Mean	17.7	20.9	0.225	
			SD	9.2	15.9		
	Euglena 3,000 mg	20	Mean	21.8	21.3	0.886	
			SD	20.5	17.4		
ALP (U/L)	Placebo	18	Mean	180.6	188.5	0.253	100 - 325
			SD	60.4	64.3		
	Euglena 500 mg	19	Mean	209.1	219.6	0.081	
			SD	56.4	58.6		
	Euglena 1,000 mg	20	Mean	207.1	212.5	0.356	
			SD	63.7	65.4		
	Euglena 3,000 mg	20	Mean	188.7	195.4	0.212	
			SD	60.1	68.2		

LD(LDH) (U/L)	Placebo	18	Mean	176.4	170.7	0.100	120 - 240
			SD	17.5	18.9		
	Euglena 500 mg	19	Mean	178.5	177.6	0.763	
			SD	23.8	25.6		
	Euglena 1,000 mg	20	Mean	174.5	177.1	0.490	
			SD	24.2	31.1		
	Euglena 3,000 mg	20	Mean	186.1	186.9	0.832	
			SD	28.9	31.3		
γ-GT (U/L)	Placebo	18	Mean	28.4	31.0	0.335	male : ≦80 female : ≦30
			SD	22.0	26.2		
	Euglena 500 mg	19	Mean	20.8	22.5	0.434	
			SD	8.7	11.1		
	Euglena 1,000 mg	20	Mean	21.7	22.5	0.542	
			SD	12.2	12.3		
	Euglena 3,000 mg	20	Mean	24.5	26.3	0.426	
			SD	14.8	17.0		
Serum Amylase (U/L)	Placebo	18	Mean	72.2	77.0	0.027	40 - 122
			SD	21.3	26.5		
	Euglena 500 mg	19	Mean	69.5	76.6	0.003	
			SD	18.1	21.4		
	Euglena 1,000 mg	20	Mean	79.3	85.6	0.024	
			SD	23.9	26.6		
	Euglena 3,000 mg	20	Mean	74.8	80.6	0.000	
			SD	18.1	21.0		
CK (U/L)	Placebo	18	Mean	113.1	104.3	0.186	male : 60 - 270 female : 40 - 150
			SD	62.5	56.9		
	Euglena 500 mg	19	Mean	95.3	115.2	0.279	
			SD	40.7	90.5		
	Euglena 1,000 mg	20	Mean	99.4	113.4	0.138	
			SD	45.7	55.5		
	Euglena 3,000 mg	20	Mean	111.4	149.9	0.324	
			SD	55.3	196.5		
Creatinine (mg/dL)	Placebo	18	Mean	0.7	0.7	0.281	male : 0.61 - 1.04 female : 0.47 - 0.79
			SD	0.1	0.1		
	Euglena 500 mg	19	Mean	0.7	0.7	0.068	
			SD	0.2	0.1		
	Euglena 1,000 mg	20	Mean	0.7	0.7	0.804	
			SD	0.1	0.2		
	Euglena 3,000 mg	20	Mean	0.7	0.7	0.257	
			SD	0.1	0.1		
Urea Nitrogen-BUN (mg/dL)	Placebo	18	Mean	12.1	11.8	0.548	8.0 - 20.0
			SD	3.4	3.0		
	Euglena 500 mg	19	Mean	11.0	12.4	0.116	
			SD	3.2	4.5		
	Euglena 1,000 mg	20	Mean	12.6	11.6	0.159	
			SD	3.7	3.9		
	Euglena 3,000 mg	20	Mean	12.7	12.1	0.276	
			SD	3.2	3.2		
Uric Acid (mg/dL)	Placebo	18	Mean	4.5	4.7	0.114	male : 3.8 - 7.0 female : 2.5 - 7.0
			SD	1.3	1.3		
	Euglena 500 mg	19	Mean	5.1	5.1	0.654	
			SD	1.4	1.5		
	Euglena 1,000 mg	20	Mean	4.7	4.6	0.416	
			SD	1.2	1.2		
	Euglena 3,000 mg	20	Mean	5.3	5.1	0.342	
			SD	1.2	1.1		

Blood Glucose (mg/dL)	Placebo	18	Mean	85.1	88.5	0.045	70 - 109
			SD	6.9	9.6		
	Euglena 500 mg	19	Mean	82.7	83.6	0.746	
			SD	10.1	12.6		
	Euglena 1,000 mg	20	Mean	83.3	86.7	0.180	
			SD	4.3	10.9		
	Euglena 3,000 mg	20	Mean	83.8	81.8	0.303	
			SD	9.8	7.9		
Total Cholesterol (mg/dL)	Placebo	18	Mean	221.1	223.3	0.610	120 - 219
			SD	37.8	30.3		
	Euglena 500 mg	19	Mean	198.4	204.7	0.242	
			SD	31.0	26.9		
	Euglena 1,000 mg	20	Mean	207.7	208.1	0.912	
			SD	33.5	29.8		
	Euglena 3,000 mg	20	Mean	212.8	209.6	0.409	
			SD	33.4	33.2		
HDL(HDL Cholesterol) (mg/dL)	Placebo	18	Mean	69.0	70.3	0.445	male : 40 - 85<br>female : 40 - 95
			SD	17.2	18.5		
	Euglena 500 mg	19	Mean	59.5	65.6	0.010	
			SD	20.0	20.3		
	Euglena 1,000 mg	20	Mean	61.7	65.8	0.029	
			SD	13.7	18.1		
	Euglena 3,000 mg	20	Mean	61.8	64.7	0.030	
			SD	14.6	14.5		
LDL(LDL Cholesterol) (mg/dL)	Placebo	18	Mean	130.7	130.9	0.937	65 - 139
			SD	35.1	29.0		
	Euglena 500 mg	19	Mean	118.3	119.3	0.830	
			SD	29.9	25.7		
	Euglena 1,000 mg	20	Mean	126.4	120.6	0.141	
			SD	31.9	26.5		
	Euglena 3,000 mg	20	Mean	131.9	127.4	0.223	
			SD	30.0	30.0		
TG(Neutral Fats) (mg/dL)	Placebo	18	Mean	116.8	115.8	0.959	30 - 149
			SD	82.5	81.7		
	Euglena 500 mg	19	Mean	106.8	93.8	0.252	
			SD	72.7	60.6		
	Euglena 1,000 mg	20	Mean	102.5	152.9	0.386	
			SD	49.9	270.5		
	Euglena 3,000 mg	20	Mean	93.4	86.2	0.070	
			SD	40.2	37.0		
Na (mEq/L)	Placebo	18	Mean	140.1	140.8	0.210	137 - 147
			SD	1.7	2.0		
	Euglena 500 mg	19	Mean	140.8	141.1	0.561	
			SD	1.9	1.7		
	Euglena 1,000 mg	20	Mean	140.5	140.7	0.724	
			SD	1.3	1.6		
	Euglena 3,000 mg	20	Mean	141.3	141.3	0.895	
			SD	1.7	1.7		
K (mEq/L)	Placebo	18	Mean	4.0	4.1	0.387	3.5 - 5.0
			SD	0.2	0.3		
	Euglena 500 mg	19	Mean	3.9	4.0	0.432	
			SD	0.2	0.3		
	Euglena 1,000 mg	20	Mean	4.0	4.0	0.600	
			SD	0.3	0.2		
	Euglena 3,000 mg	20	Mean	4.1	4.0	0.080	
			SD	0.2	0.2		

Cl (mEq/L)	Placebo	18	Mean	103.9	104.5	0.219	98 - 108
			SD	1.5	1.8		
	Euglena 500 mg	19	Mean	103.1	103.5	0.420	
			SD	2.1	2.3		
	Euglena 1,000 mg	20	Mean	103.4	104.0	0.091	
			SD	1.8	1.8		
	Euglena 3,000 mg	20	Mean	104.1	104.9	0.183	
			SD	1.9	2.0		
Ca (mg/dL)	Placebo	18	Mean	9.1	9.2	0.273	8.4 - 10.4
			SD	0.3	0.4		
	Euglena 500 mg	19	Mean	9.2	9.5	0.009	
			SD	0.3	0.4		
	Euglena 1,000 mg	20	Mean	9.3	9.3	0.782	
			SD	0.3	0.3		
	Euglena 3,000 mg	20	Mean	9.2	9.2	0.592	
			SD	0.3	0.3		
Mg (mg/dL)	Placebo	18	Mean	2.1	2.1	0.001	1.9 - 2.5
			SD	0.1	0.1		
	Euglena 500 mg	19	Mean	2.2	2.1	0.001	
			SD	0.2	0.1		
	Euglena 1,000 mg	20	Mean	2.2	2.1	0.131	
			SD	0.1	0.1		
	Euglena 3,000 mg	20	Mean	2.1	2.1	0.012	
			SD	0.1	0.2		
Fe (μg/dL)	Placebo	18	Mean	108.1	97.4	0.297	male : 50 - 200<br>female : 40 - 180
			SD	45.1	43.6		
	Euglena 500 mg	19	Mean	106.5	104.4	0.843	
			SD	37.8	33.1		
	Euglena 1,000 mg	20	Mean	117.1	111.8	0.690	
			SD	40.1	31.7		
	Euglena 3,000 mg	20	Mean	113.4	92.9	0.007	
			SD	32.7	33.7		
WBC (White Blood Cell Count) (/μL)	Placebo	18	Mean	5588.9	5705.6	0.699	3300 - 9000
			SD	1200.4	1370.0		
	Euglena 500 mg	19	Mean	6431.6	6389.5	0.905	
			SD	1617.3	1965.8		
	Euglena 1,000 mg	20	Mean	6010.0	5855.0	0.712	
			SD	1380.3	1732.2		
	Euglena 3,000 mg	20	Mean	5680.0	6270.0	0.073	
			SD	1115.7	2112.9		
RBC (Red Blood Cell Count) ($\times 10^4$/μL)	Placebo	18	Mean	452.8	453.1	0.955	male : 430 - 570<br>female : 380 - 500
			SD	37.6	42.7		
	Euglena 500 mg	19	Mean	476.1	471.4	0.380	
			SD	47.9	39.5		
	Euglena 1,000 mg	20	Mean	468.2	462.1	0.150	
			SD	33.7	35.9		
	Euglena 3,000 mg	20	Mean	462.2	460.7	0.755	
			SD	33.6	39.0		
Hb(Hemoglobin) (g/dL)	Placebo	18	Mean	13.5	13.5	0.818	male : 13.5 - 17.5<br>female : 11.5 - 15.0
			SD	1.7	1.7		
	Euglena 500 mg	19	Mean	14.4	14.4	0.784	
			SD	1.4	1.1		
	Euglena 1,000 mg	20	Mean	14.3	14.1	0.104	
			SD	1.3	1.3		
	Euglena 3,000 mg	20	Mean	14.2	14.2	0.956	
			SD	1.4	1.5		

Ht(Hematocrit) (%)	Placebo	18	Mean	42.2	41.8	0.306	male : 39.7 - 52.4 female : 34.8 - 45.0
			SD	4.0	4.4		
	Euglena 500 mg	19	Mean	44.7	43.9	0.192	
			SD	4.0	2.9		
	Euglena 1,000 mg	20	Mean	44.1	43.1	0.022	
			SD	3.1	3.4		
	Euglena 3,000 mg	20	Mean	43.8	43.4	0.395	
			SD	3.5	3.9		
MCV (fL)	Placebo	18	Mean	93.3	92.4	0.033	85 - 102
			SD	6.1	6.0		
	Euglena 500 mg	19	Mean	94.1	93.3	0.114	
			SD	5.2	4.2		
	Euglena 1,000 mg	20	Mean	94.4	93.4	0.076	
			SD	3.6	4.4		
	Euglena 3,000 mg	20	Mean	94.8	94.2	0.281	
			SD	3.7	3.8		
MCH (pg)	Placebo	18	Mean	29.8	29.7	0.690	28.0 - 34.0
			SD	2.7	2.6		
	Euglena 500 mg	19	Mean	30.4	30.6	0.145	
			SD	1.9	1.5		
	Euglena 1,000 mg	20	Mean	30.5	30.5	0.856	
			SD	1.5	1.4		
	Euglena 3,000 mg	20	Mean	30.8	30.8	0.661	
			SD	1.7	1.4		
MCHC (%)	Placebo	18	Mean	31.9	32.1	0.172	30.2 - 35.1
			SD	1.4	1.0		
	Euglena 500 mg	19	Mean	32.3	32.8	0.004	
			SD	0.8	0.7		
	Euglena 1,000 mg	20	Mean	32.3	32.7	0.103	
			SD	1.1	1.1		
	Euglena 3,000 mg	20	Mean	32.4	32.7	0.126	
			SD	1.0	0.8		
PLT (Platelet Count) ($\times 10^4/\mu L$)	Placebo	18	Mean	27.6	29.5	0.021	14.0 - 34.0
			SD	3.9	4.5		
	Euglena 500 mg	19	Mean	30.8	31.9	0.021	
			SD	5.2	6.3		
	Euglena 1,000 mg	20	Mean	26.6	26.1	0.335	
			SD	5.2	4.9		
	Euglena 3,000 mg	20	Mean	27.0	27.0	0.878	
			SD	4.6	4.6		

Supplemental Table 2: Summary of blood pressure and pulse rate test results. Weeks 0, 4, 8, and 12 measurements and significant differences with corresponding paired t-tests within each group are shown. The investigator found no problems.

Parameter	Group	n		0 weeks	4 weeks	8 weeks	12 weeks
Pulse Rate (bpm)	Placebo	18	Mean	67.3	70.2	70.0	70.4
			SD	7.0	10.6	11.8	12.1
			p -Value		0.460	0.776	0.690
	Euglena 500 mg	19	Mean	72.3	74.1	73.7	72.8
			SD	8.4	12.7	9.6	8.4
			p -Value		1.000	1.000	1.000
	Euglena 1,000 mg	20	Mean	72.7	68.2	69.6	72.8
			SD	10.8	9.0	10.4	16.3
			p -Value		0.022	0.214	1.000
	Euglena 3,000 mg	20	Mean	70.0	70.2	69.9	69.9
			SD	8.4	10.2	7.6	9.6
			p -Value		1.000	1.000	1.000
SBP(Systolic Blood Pressure) (mmHg)	Placebo	18	Mean	109.8	112.9	110.1	113.0
			SD	8.4	10.1	11.3	7.7
			p -Value		0.515	1.000	0.194
	Euglena 500 mg	19	Mean	119.7	117.1	118.3	120.1
			SD	15.7	16.5	14.5	14.1
			p -Value		1.000	1.000	1.000
	Euglena 1,000 mg	20	Mean	113.2	113.1	113.3	115.7
			SD	11.3	13.4	12.2	14.9
			p -Value		1.000	1.000	0.888
	Euglena 3,000 mg	20	Mean	114.3	112.5	113.0	114.6
			SD	10.2	12.1	11.3	14.8
			p -Value		1.000	1.000	1.000
DBP(Diastolic Blood Pressure) (mmHg)	Placebo	18	Mean	67.1	66.3	66.9	68.9
			SD	7.5	8.9	7.9	8.4
			p -Value		1.000	1.000	0.683
	Euglena 500 mg	19	Mean	74.4	71.7	70.3	74.2
			SD	11.0	10.6	10.0	9.9
			p -Value		0.413	0.054	1.000
	Euglena 1,000 mg	20	Mean	74.8	68.8	68.7	71.1
			SD	8.0	8.8	8.8	10.1
			p -Value		0.028	0.021	0.311
	Euglena 3,000 mg	20	Mean	73.5	68.6	68.4	71.4
			SD	7.6	9.4	9.3	9.9
			p -Value		0.064	0.013	0.817
